# A Data-Driven Approach for Leveraging Inline and Offline Data to Determine the Causes of Monoclonal Antibody Productivity Reduction in the Commercial-Scale Cell Culture Process

**DOI:** 10.3390/pharmaceutics16081082

**Published:** 2024-08-17

**Authors:** Sheng Zhang, Hang Chen, Yuxiang Wan, Haibin Wang, Haibin Qu

**Affiliations:** 1 Pharmaceutical Informatics Institute, College of Pharmaceutical Sciences, Zhejiang University, Hangzhou 310058, China; zsheng@zju.edu.cn (S.Z.); chen_hang@zju.edu.cn (H.C.); 2 BioRay Pharmaceutical Co., Ltd., Taizhou 318000, China; yxwan@bioraypharm.com (Y.W.); hbwang@bioraypharm.com (H.W.)

**Keywords:** cell culture, data-driven, exploratory data analysis, functional data analysis, contrastive principal component analysis, root cause analysis

## Abstract

The monoclonal antibody (mAb) manufacturing process comes with high profits and high costs, and thus mAb productivity is of vital importance. However, many factors can impact the cell culture process, and lead to mAb productivity reduction. Nowadays, the biopharma industry is actively employing manufacturing information systems, which enable the integration of both online data and offline data. Although the volume of data is large, related data mining studies for mAb productivity improvement are rare. Therefore, a data-driven approach is proposed in this study to leverage both the inline and offline data of the cell culture process to discover the causes of mAb productivity reduction. The approach consists of four steps, namely data preprocessing, phase division, feature extraction and fusion, and cluster comparing. First, data quality issues are solved during the data preprocessing step. Next, the inline data are divided into several phases based on the moving window *k*-nearest neighbor method. Then, the inline data features are extracted via functional data analysis and combined with the offline data features. Finally, the causes of mAb productivity reduction are identified using the contrasting clusters via the principal component analysis method. A commercial-scale cell culture process case study is provided in this research to verify the effectiveness of the approach. Data from 35 batches were collected, and each batch contained nine inline variables and seven offline variables. The causes of mAb productivity reduction were identified to be the lack of nutrients, and recommended actions were taken according to the result, which was subsequently proven by six validation batches.

## 1. Introduction

Biopharmaceuticals are therapeutic drugs derived from biological sources, such as vaccines and monoclonal antibodies (mAbs). Biopharmaceuticals are usually used to cure cancer and autoimmune diseases, collectively forming a substantial market. The global sales of biopharmaceuticals reached USD 343 billion in 2021, with 63.3% of these sales coming from mAbs [1]. In 2022, six out of ten top drugs according to sales were mAbs, each surpassing USD 10 billion [2], and sales of the best-selling mAb, adalimumab (under the brand name Humira), produced by AbbVie, amounted to USD 21.6 billion. It can be forecasted that the market size of mAbs will also experience high growth in the next 5 years [3]. Meanwhile, the manufacturing of mAbs is costly in terms of both time and materials, e.g., one batch usually lasts for more than 20 days and the material costs are over hundreds of thousands of dollars. Given the high profits and the high costs, the productivity of mAbs is a key performance indicator of the manufacturing process.

The manufacturing of mAbs is generally undertaken in fed-batch mammal cell culture on the commercial scale, and the commonly used cell system is the Chinese hamster ovary (CHO) cell line [4]. In a typical cell culture process, the cells are firstly cultured at a laboratory scale (e.g., 125 mL to 5 L) and then gradually transferred to a series of bioreactors from the small scale (e.g., 20 L) to the production scale (e.g., 2000 L) [5]. During the production-scale process, additional nutrients (including glucose, amino acids, and other necessary components) are fed to bioreactors to support cell growth and protein expression.

Due to the intrinsic complexities of cell culture, the mAb productivity of each batch may not be guaranteed to be at a high level. Optimizing the cell culture process to produce more mAbs is not an easy task because many factors will affect productivity. First of all, the cell line and culture medium are essential to mAb manufacturing [6], but typically, they are usually fixed for commercial-scale manufacturing because such major modifications are considered post-approval changes and must be approved by regulatory agencies. Second, process parameters, including temperature [7], pH [8], partial pressure of CO_2_ [9], dissolved O_2_ (DO) [8], and other process parameters [10,11], are also important for cell growth and protein expression. Third, process control strategies have a significant impact on the cell culture process. For instance, improving how nutrients are fed [12,13] and how air sparging works [14] has been proven to be beneficial to mAb productivity and quality. Therefore, it is difficult for biopharma companies to identify the causes of mAb productivity reduction and to optimize operations for higher yield.

To tackle the aforementioned problems, the bioprocess has been embracing innovative tools, such as process analytical technology [15,16], mechanistic modeling [17,18,19], data-driven modeling [20,21], and digital twins [22,23,24]. However, mechanistic modeling and digital twins require solid knowledge of biological mechanisms [23], but biopharma companies usually lack such knowledge. Mechanistic models developed by small-scale data also need to be carefully tuned before being applied to commercial-scale processes, and such difficulties hinder the potential to integrate the tools into large-scale cell culture processes. In comparison, data-driven modeling does not require profound process mechanisms and has the potential to improve mAb manufacturing.

Nowadays, the biopharma industry is actively employing manufacturing information systems, such as the supervisory control and data acquisition (SCADA) system, the manufacturing execution system (MES), and the laboratory information management system (LIMS). These systems enable the collection and integration of a large amount of cell culture process data, including offline data and inline data [25]. There are some investigations into how to analyze cell culture process data. Goldrick et al. outlined a multivariate data analysis methodology to quantify the mAb product equivalence of different batches at a large scale, and to predict the viable cell concentration at a small scale [26]. Sokolov et al. collected mAb quality data at four different scales (from 0.5 mL to 280 L) and used algorithms like principal components analysis (PCA), partial least square (PLS), and decision tree to fully analyze the data for decision-making at each stage of cell culture process development [27]. Manapragada et al. applied the artificial neural network to model the cell culture process, with amino acids fed as the input and the product yield as the output to solve the feed optimization problem [20]. The above studies are all based on offline data, while investigations of inline data are somewhat rare. Le et al. employed support vector regression and PLS to predict the mAb concentration and the final lactate concentration based on both offline and inline data [28]. Bayrak et al. developed a real-time product prediction framework based on various machine learning algorithms [29]. Besides product prediction, a fault classification and diagnosis approach to the cell culture process has been proposed based on inline data to identify the causes of the low-process-performance process [30]. In summary, the current research mainly focuses on offline data analysis and existing research on inline data analysis to build product prediction models. However, there are few studies on how to utilize both inline and offline data to optimize cell culture processes for productivity improvement.

In this study, a data-driven approach is proposed to exploit the manufacturing data on the commercial-scale cell culture process to help improve the mAb productivity. This approach fully utilized the inline and offline data on both high-productivity and low-productivity batches. The inline data are first fed into the preprocessing step to ensure high quality, then divided into several operating phases using the moving window *k*-nearest neighbor (*k*NN) rule [31]. After phase division, the features of inline data are extracted via functional data analysis (FDA) [32], which is combined with offline data to form an extended feature matrix. The contrasting clusters in the PCA (ccPCA) method [33] are applied to the extended feature matrix to identify the major differences between high-productivity and low-productivity batches, thus revealing the causes of productivity reduction. Finally, practical decisions are made according to the previous analysis results. A case study containing data from a commercial-scale cell culture process is considered in order to prove the effectiveness of the proposed approach.

The main contributions of this work are listed below:

- A data-driven approach is proposed to leverage both the inline and offline data to identify the causes of mAb productivity reduction.
- The features of inline data are extracted using the FDA method and then fused with offline data to obtain an extended feature matrix to represent the cell culture process. The causes of mAb productivity are analyzed via the ccPCA method based on the extended feature matrix.
- A commercial-scale case study is provided to verify the effectiveness of the proposed approach.

## 2. Materials and Methods

### 2.1. Preliminaries

#### 2.1.1. *k*NN

The *k*NN method is a widely used machine learning algorithm for classification tasks. An unknown sample is labeled depending on its distance to the nearest training neighbor. Given a matrix, $X={\left[x_{1},x_{2},\ldots ,x_{n}\right]}^{T}\in R^{n\times J}$, where *n* is the number of samples, and *J* is the number of process variables, the distance between $x_{i}$ and $x_{j}$ is calculated via Equation (1). To build the *k*NN set {$x_{i,1},x_{i,2},\ldots ,x_{i,k}$} of a certain sample, $x_{i}$, the rest of the *n* − 1 samples are sorted by distance in ascending order, and the first *k* samples are chosen as the *k*NN set.

(1) $$d(x_{i},x_{j})=\left\|x_{i}-x_{j}\right\|$$

#### 2.1.2. FDA

The process data of the *i*th batch are organized into a 2D matrix, $X_{i}\left(J\times K_{i}\right)$, where *J* is the number of process variables and $K_{i}$ is the batch length (i.e., the number of sampling points). In many cases, different batches have different lengths, that is, the values of $K_{i}$ of each batch are not always equal. This introduces the uneven batch length problem, and many traditional process monitoring methods do not apply to this problem, such as multiway PCA [34]. Therefore, time warping techniques, like dynamic time warping (DTW) [35], are applied to align the batches for even batch lengths. However, such techniques are dynamic programming methods, which often require huge computation resources, especially when the batch length is very long, as in the cell culture process. Furthermore, the trajectories after DTW may not be consistent with the original trajectories, thus misrepresenting the process characteristics.

Unlike DTW, the FDA method tries to approximate the whole process trajectory of a variable in a batch using basis functions [36], such as in Equation (2), where $x_{i,j}$ is the process data vector of the *j*th variable in the *i*th batch, $\phi_{i,j,m}\left(t\right)$ is the *m*th basis function of the *j*th variable in the ith batch, $c_{i,j,m}$ is the corresponding coefficient of $\phi_{i,j,m}\left(t\right)$, and $M_{j}$ is the number of basis functions. After FDA approximation, the original discrete process data are converted into smooth functions.

(2) $$x_{i,j}=\sum_{m=1}^{M_{j}}{c_{i,j,m}\phi }_{i,j,m}\left(t\right)$$

Typical basis functions include the Fourier basis for periodic data, the B-spline basis for non-periodic data, and the polynomial basis for data without obvious functional features [32]. Different variables should be approximated using different types and numbers of basis functions according to the variable characteristics. For the *j*th variable, if the same type and the same number of basis functions are chosen across all batches, the original process data, ***X*** (with *I* batches and *J* variables), can be represented as Equation (3), where *I* is the number of batches. Consequently, the uneven batch length problem can be solved because each batch has the same number of basis functions.

(3) $$X=\left[\begin{array}{cccc} \sum_{m=1}^{M_{1}}{c_{1,1,m}\phi }_{1,1,m}\left(t\right) & \sum_{m=1}^{M_{2}}{c_{1,2,m}\phi }_{1,2,m}\left(t\right) & \cdots & \sum_{m=1}^{M_{J}}{c_{1,J,m}\phi }_{1,J,m}\left(t\right)\\ \sum_{m=1}^{M_{1}}{c_{2,1,m}\phi }_{2,1,m}\left(t\right) & \sum_{m=1}^{M_{2}}{c_{2,2,m}\phi }_{2,2,m}\left(t\right) & \cdots & \sum_{m=1}^{M_{J}}{c_{2,J,m}\phi }_{2,J,m}\left(t\right)\\ \vdots & \ldots & \ddots & \vdots \\ \sum_{m=1}^{M_{1}}{c_{I,1,m}\phi }_{I,1,m}\left(t\right) & \sum_{m=1}^{M_{2}}{c_{I,2,m}\phi }_{I,2,m}\left(t\right) & \cdots & \sum_{m=1}^{M_{J}}{c_{I,J,m}\phi }_{I,J,m}\left(t\right) \end{array}\right]$$

Since each variable has the same type and the same number of basis functions, the process variable characteristics can be represented by the basis function coefficients. The number of basis function coefficients for each batch is $\sum_{i=j}^{J}M_{j}$, so the size of the coefficient matrix, ***C***, of all batches (shown in Equation (4)) is $I\times \sum_{i=j}^{J}M_{j}$.

(4) $$C=\left[\begin{array}{cccc} c_{1,1} & c_{1,2} & \cdots & c_{1,J}\\ c_{2,1} & c_{2,2} & \cdots & c_{2,J}\\ \vdots & \ldots & \ddots & \vdots \\ c_{I,1} & c_{I,2} & \cdots & c_{I,J} \end{array}\right]\;c_{i,j}=[c_{i,j,1},c_{i,j,2},\ldots ,c_{i,j,M_{j}}]$$

**Figure 1:**
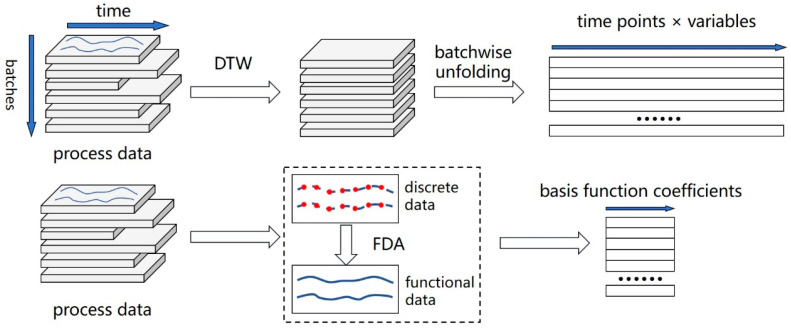
The schemes of DTW and FDA. Data after DTW are stretched or compressed to have the same length and then unfolded batch-wisely for further analysis; data after FDA are approximated using basis functions and the coefficients are extracted as features.

Now, the original uneven process data matrix, X, is transformed into the coefficient matrix, ***C***, and the basis function coefficients are treated as the features of the process data. The schemes of DTW and FDA are shown in Figure 1.

#### 2.1.3. ccPCA

ccPCA is an analysis method designed to measure the contribution of each feature to one cluster’s contrast to the others. In other words, ccPCA can identify the features that contribute most to the differences between high-productivity and low-productivity batches, and such features may be highly related to the root causes of productivity reduction. It has been proven that ccPCA can identify the root causes of evaporation performance degradation in the field of herbal medicine manufacturing [37], and in this study, the method is introduced to biopharma manufacturing.

ccPCA is based on contrastive PCA (cPCA), which is developed to find the patterns that are enriched in one dataset relative to another dataset [38]. Assuming ***X*** is the dataset of interest (target cluster, e.g., the high-productivity batch data) and ***Y*** is the comparison dataset (background cluster, e.g., the low-productivity batch data), cPCA tries to find the direction, $v^{*}$, in which the variance matrix of ***X*** is high but the covariance of ***Y*** is low. Let $C_{X}$ be the covariance matrix of ***X***, $C_{Y}$ be the covariance matrix of ***Y***, and $\lambda_{X}\left(v\right)≝v^{T}C_{X}v$, and $\lambda_{Y}\left(v\right)≝v^{T}C_{Y}v$; $v^{*}$ is found by solving Equation (5):

(5) $$v^{*}=\arg\underset{v}{\max}\lambda_{X}\left(v\right)-\alpha \lambda_{Y}\left(v\right)=\arg\underset{v}{\max}v^{T}(C_{X}-\alpha C_{Y})v$$

where α is the contrastive parameter (α≥0). If α=0, cPCA deteriorates to classical PCA, only maximizing the variance of the target cluster. $v^{*}$ is the first eigenvector of $C_{X}-\alpha C_{Y}$, which is also called the first contrastive principal component (cPC).

However, cPCA aims to discover the patterns within the target cluster (e.g., the differences among the high-productivity batches), but ignore the differences between the target and the background clusters. Consequently, it is not suitable for cPCA to find the differences between high-productivity and low-productivity batches. This issue is solved via ccPCA by taking the union set as the target cluster, ***X***, and the data, other than the original target cluster, as the background cluster, ***Y***, in Equation (5). Therefore, the optimizing problem can be rewritten as follows:

(6) $$v^{*}=\arg\underset{v}{\max}\lambda_{E}\left(v\right)-\alpha \lambda_{R}\left(v\right)=\arg\underset{v}{\max}v^{T}(C_{E}-\alpha C_{R})v$$

where ***E*** is the entire dataset (the union set of both the target and background dataset), ***R*** is the background dataset (R=E\X), $C_{E}$ is the covariance matrix of ***E***, and $C_{R}$ is the covariance matrix of ***R***. After this step, the target cluster can be separated from the background, and the loadings of the first cPC ($v^{*}$) are taken as contributions to the differences between the target and the background cluster.

### 2.2. Proposed Approach

The proposed approach is developed for both inline and offline data analysis to identify the causes of mAb productivity reduction in the cell culture process. In this study, the inline data refer to the real-time data generated by the sensors equipped in the bioreactors, such as the real-time temperature, pH, and DO. The offline data are generated by offline sample analysis, usually including the viable cell density (VCD), the cell viability, and the concentrations of some important nutrients and metabolites. The major difference between the inline and offline data is the measurement period. The measurement period of the inline data is very short, usually seconds or minutes, while the measurement period of the offline data typically lasts days.

**Figure 2:**
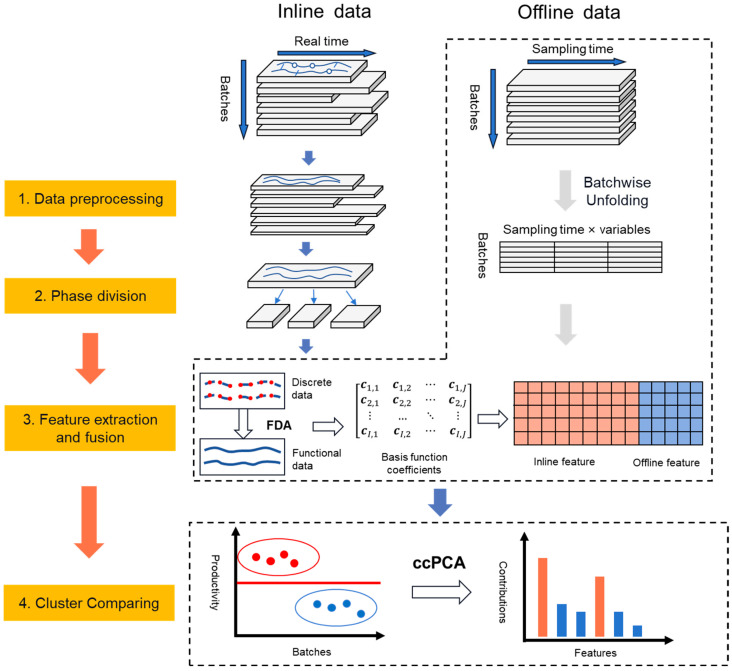
A flowchart of the proposed approach for the data analysis of the cell culture process.

The proposed approach comprises 4 steps, namely data preprocessing, phase division, feature extraction and fusion, and cluster comparison. A flowchart of the approach is shown in Figure 2.

#### 2.2.1. Data Preprocessing

The data preprocessing step aims to enhance the data quality. The offline data are typically generated using analysis equipment and are carefully checked by the operators. Therefore, the offline data are usually of high quality, and the data preprocessing step is designed to improve the inline data quality. The inline data are generated by sensors automatically and transmitted to the real-time database through networks. The data quality issues mainly include the 3 types listed below:

1. Missing data caused by network failures: Network failures disconnect the equipment and the real-time database; thus, the values during network downtime are not recorded. The amount of missing data depends on how long it takes to solve network failures.
2. Data spikes caused by sensor failures: Data spikes, although not often seen, manifest as a sudden drop or rise in normal data that fall out of the reasonable ranges (e.g., the pH drops to 0 or the temperature rises to 100 °C during the culture process), and can be easily identified by simple rules.
3. Variables with little information: There are many sensors equipped in both bioreactors and their accessories, but the variables not directly linked to the bioreactors may not reflect the dynamic characteristics of the cell culture process. Furthermore, some process variables are stationary across the whole batch, and their variances are zero or very low. Such variables are considered to have little contribution to the data analysis of the cell culture process.

**Figure 3:**
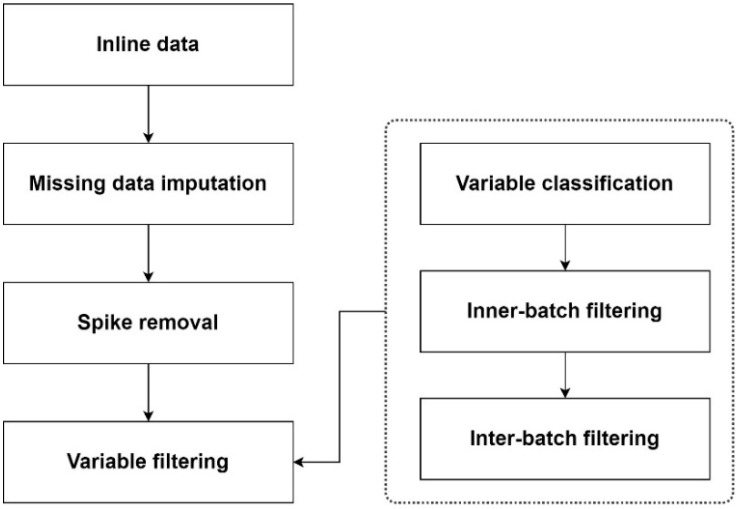
A flowchart of the data preprocessing step.

The data preprocessing step shown in Figure 3 is aimed at solving the aforementioned data quality issues. First, the amount of missing data is evaluated. If the amount is too large (e.g, over 30%), the whole batch would be discarded; if the missing data only amount to a small portion (e.g., the occurrence of missing data lasts only 1 min in several hours), the missing data points are imputed by the mean values of the neighboring data points. Second, the data spikes are also replaced by the mean values. Third, the variables with little information are filtered.

To identify the variables with little information, three substeps are required. Variable classification is first performed to exclude the variables that are not directly linked to the bioreactors. Then, inner-batch filtering is performed to calculate the variance in a variable in each batch (inner-batch variance). If the inner-batch variance is not very low, the variable is kept for further analysis. Finally, in the inter-batch filtering substep, if the inner-batch variance in a variable is very low, the mean value of this variable of each batch is calculated, and the variance in the mean values is considered the inter-batch variance. Similarly, if the inter-batch variance is very low, the corresponding variable is excluded.

#### 2.2.2. Phase Division

The cell culture process is composed of several phases based on the cell growing status or manual operations. For instance, additional mediums are fed to the bioreactors on different days to provide sufficient nutrients for cell growth and a temperature shift is implemented after several days for cells to express protein. After such operations, the process characteristics may not be consistent with the previous characteristics, which leads to a new process phase. The multiphase issue will make data analysis more complicated without phase division. The process can be divided by the feeding time, the temperature shift time, and other criteria. However, there is no standard method for phase division. One way to carry out phase division is to classify similar samples into the same phase. The *k*NN rule is often adopted to find similar samples from the whole batch, but this is very time-consuming if the batch length is very long. Furthermore, the similar samples determined via the *k*NN rule are only close in space but not in time, i.e., a sample at the beginning of the batch may be classified as the same as the samples at the end of the batch. To solve these problems, the moving window *k*NN is utilized in this step.

The moving window *k*NN is based on two assumptions: (1) a phase is relatively stable, and the samples in the same phase are similar; (2) the transition period between two stable phases is short and highly dynamic, where the samples are also very different from those in the stable phase. Given $x_{t}$ as the sample at time *t*, the corresponding moving window starts from $x_{t-L}$, and ends with $x_{t+L}$. The *k*NN set of $x_{t}$ is found within the moving window, and the *k*NN set distance is defined as Equation (7), where $x_{t,i}$ is the *i*th sample in the *k*NN set:

(7) $$d(x_{t})=\sqrt{\sum_{i=1}^{k}d{\left(x_{t},x_{t,i}\right)}^{2}/k}$$

If $x_{t}$ belongs to a stable phase, $d(x_{t})$ is small; otherwise, $d(x_{t})$ will become much higher. When the *k*NN set distance for each sample in a batch is computed, the transition phases are identified if $d\left(x_{t}\right)$ is larger than the threshold, δ. Via the moving window *k*NN method, each batch can be divided into the same number of stable phases, and the transition phases are discarded before subsequent analysis.

#### 2.2.3. Feature Extraction and Fusion

**Figure 4:**
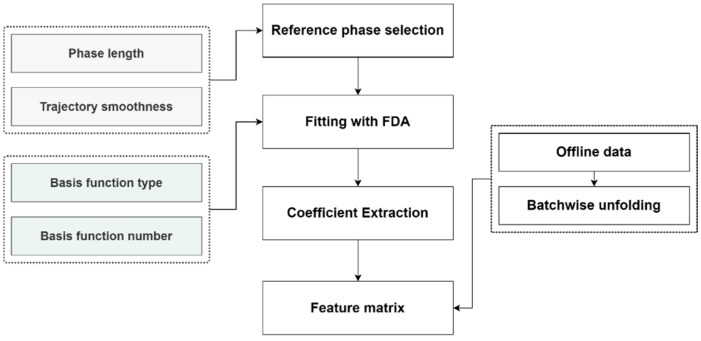
A flowchart of the feature extraction step.

Each batch is segmented into several phases after the phase division step, but the lengths of each phase in all batches are not equal, leading to the uneven batch length problem. Although some techniques like DTW can solve the problem by compressing or stretching the process trajectories, they require a lot of computation resources, and the relationship between the original data and the processed data may be distorted after DTW. The FDA method is introduced in this study to extract the inline data features, as shown in Figure 4. For the same phase in all batches, the FDA method uses the same number and types of basis functions to fit a specific process variable, so that all batches can be represented by a basis function coefficient matrix in Equation (4).

First, a reference phase is selected for all batches to determine the basis function type and number. Two parameters are combined to create an indicator for the reference phase, namely the phase length and the phase trajectory smoothness. A combined indicator, $H_{p}$, is calculated using Equation (8), where $L_{p}$ is the length of the *p*th phase, $L_{max}$ is the max batch length, $x_{p,m,t}$ is the value of the *m*th variable in the *p*th phase at time *t*, *M* is the number of process variables, $\bar{D_{p,m}^{2}}$ is the average second-order difference in the *m*th variables in the *p*th phase, and $\bar{D_{m,max}^{2}}$ is the maximum value of $\bar{D_{p,m}^{2}}$. With this indicator, the reference phase is relatively longer and rougher compared to other phases. Note that if each batch is divided into *P* phases, there are *P* reference phases to be searched in total.

(8) $$H_{p}=\left(\frac{L_{p}}{L_{max}}+\sum_{m=1}^{M}\frac{\bar{D_{p,m}^{2}}}{\bar{D_{m,max}^{2}}}\right)/(M+1)\;\bar{D_{p,m}^{2}}=\frac{\sum_{t=1}^{L_{p}-2}\left[\left(x_{p,m,t+2}-x_{p,m,t+1}\right)-\left(x_{p,m,t+1}-x_{p,m,t}\right)\right]}{L_{p}-2}$$

Second, the basis function type for each variable is determined according to the reference phase. There are 4 commonly used basis function types: the Fourier basis, the B-spline basis, the polynomial basis, and the constant basis. The Fourier basis is used to fit periodic variables, and the B-spline basis is typically used to fit non-periodic variables with complex smooth shapes. The polynomial basis is used to fit variables behaving like simple curves such as the third-order curve [32]. After the basis function type is selected, the number of the B-spline basis functions is optimized by the mean squared error (MSE) between the fitted data and the original data. When the MSE does not decrease sharply with the increase in the basis function number, the proper number is then found.

Third, after the previous substeps, the original data are fitted by basis functions. Given a dataset containing *I* batches and *J* variables, with each batch divided into *P* phases, the total number of basis functions used, $M_{f}$, is calculated in Equation (9):

(9) $$M_{f}=\sum_{i=1}^{I}\sum_{p=1}^{P}\sum_{j=1}^{J}M_{i,p,j}$$

where $M_{i,p,j}$ is the basis function number for the *j*th variable in the *p*th phase of the *i*th batch. The coefficient of each basis function is then extracted to form a 2D matrix measuring a size of $I\times \sum_{p=1}^{P}\sum_{j=1}^{J}M_{i,p,j}$. It is noteworthy that the number and type of basis functions are the same for a specific variable in different batches, and only with this can the original data be represented by the coefficient matrix.

Fourth, the offline data are unfolded batch-wisely into a matrix measuring a size of I×S; then, the offline data matrix and the basis function coefficient matrix are concatenated to form an extended feature matrix measuring a size of $I\times (S+\sum_{p=1}^{P}\sum_{j=1}^{J}M_{i,p,j}$) for further analysis.

#### 2.2.4. Cluster Comparing

The core of the proposed approach is to analyze the differences between high-productivity and low-productivity batches to find the causes of productivity reduction. In this step, the high-productivity and low-productivity batches are labeled as different clusters and ccPCA is utilized to calculate the contribution of each variable to the differences. Before building the ccPCA model, the entire feature matrix containing both the high-productivity and low-productivity batches is taken as ***E***, and the feature matrix of low-productivity batches is taken as ***R*** in Equation (5). Then, the ccPCA model is developed to compare the cluster differences, and the contributions of variables are calculated using the loading vector of the first cPC. The high contribution of variables is strongly related to the causes of productivity reduction. Once the causes of productivity reduction are discovered, decisions for future manufacturing are carefully made in order to improve mAb production.

## 3. Results and Discussion

### 3.1. The Cell Culture Process

#### 3.1.1. Process Description

**Figure 5:**
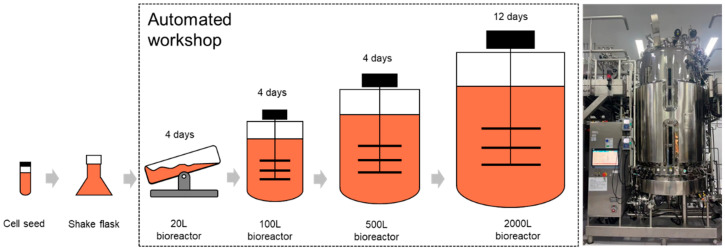
A scheme of the cell culture process. The CHO cells are cultured at different scales from 125 mL to 2000 L. A picture of the 2000 L bioreactor is shown on the right.

In this study, a case study of a commercial-scale cell culture process is introduced to validate the feasibility of the proposed approach. The process is derived from a Chinese biopharma factory, which was designed to manufacture a product biosimilar to Adalimumab. The scheme of the cell culture process is shown in Figure 5. The CHO cell seeds are firstly cultured at a small, laboratory scale (125 mL), and then transferred to shake flasks at lightly larger scales (from 500 mL to 5 L). During this culture stage, the operations are manual and last for 15 days. After culture at the shake flask scale, the cells are transferred to the bioreactors at industrial scales in an automated workshop for 24 days. Therefore, a batch of the cell culture process lasts 3 days in total. In the automated workshop, four different scales of bioreactors are employed to culture cells sequentially, namely 20 L, 100 L, 500 L, and 2000 L. In the first three bioreactors, the cells grow quickly and have a large VCD. In the last bioreactor (2000 L), the cells are supposed to grow for 2 days, and then the temperature and pH settings are changed to turn the cells from the growing stage to the protein expression stage for another 10 days. In other words, the cell culture process conducted in the 2000 L bioreactor is of vital importance for mAb production. During this process, additional glucose and cell culture medium is fed to the bioreactor to provide adequate nutrients for cells to grow and express mAbs. The additional glucose is fed daily and the additional medium is fed on Day 2, 4, 6, and 8.

The process problem was that mAb production decreased to a low level, which directly led to a profit reduction. The process experts and operators were not aware of the causes and did not have a deep process understanding to make fast and effective decisions to improve mAB productivity.

#### 3.1.2. Process Data

The automated workshop deployed the SCADA system, which enables inline data collection. In this case, both the inline data and offline data of the 2000 L bioreactor during the period 2022–2023 were collected. There were 35 batches in total collected for data analysis, including 6 validation batches. The offline data contained seven variables, which were measured once a day for 12 days; therefore, the offline data was composed of a 3D matrix measuring a size of 35 × 7 × 12. The inline data contained 71 variables, and the sampling interval was 10 s, i.e., there were 613,440 (71 × 24 × 60 × 60 ÷ 10) sampling points in one day, and about 7,361,280 sampling points in total in one batch (12 days). Among the 71 variables, there were 51 measurement variables, 14 setting variables, and 6 process status variables. The inline data were stored in a real-time database (ESP-iSYS, Zhejiang SUPCON Technology Co., Ltd., Hangzhou, China) through the OPC protocol.

### 3.2. Data Preprocessing Result

The inline data quality was evaluated after data collection. The missing values were first checked. There were three batches in which a large proportion of missing values existed, and thus, these three batches were discarded. Among the 71 variables, 8 variables had many missing values (the missing value proportion was over 30%). Similarly, these variables were also excluded. Then, the data spikes observed were replaced by the mean values of their neighboring values.

Variable filtering was conducted after solving the missing value and data spike issues. Among the 63 variables, 6 variables were related to the cleaning in place (CIP) operation, and 19 variables were the temperatures in different pipelines. Only 26 variables were directly linked to the bioreactor, among which there were 15 measurement variables and 11 setting point variables. The inner-batch and inter-batch variances of these 26 variables were calculated, and 9 measurement variables were adopted for further data analysis. The filtered inline variables and the offline variables are listed in Table 1. There are two pH and DO sensors in one bioreactor, and they are named as pH1, pH2, DO1 and DO2.

In summary, the dataset after data preprocessing contained 32 batches, and each batch had 16 variables. Since the dataset is confidential, the details of each variable are not publicly available.

### 3.3. Phase Division Result

The moving window *k*NN rule was applied to find similar samples and to calculate the *k*NN set distance for phase division. In this case, the moving window length was set to 1200 sampling points (200 min), and *k* was set to 300 sampling points (50 min). That is, given a sample, $x_{t}$, and its moving window, $\left[x_{t-1200},x_{t+1200}\right]$, the method found 300 samples similar to $x_{t}$ to form a *k*NN set, and samples in the *k*NN set covered 50 min. Then, the *k*NN set distance $d(x_{t})$ was calculated according to the 300 samples.

**Figure 6:**
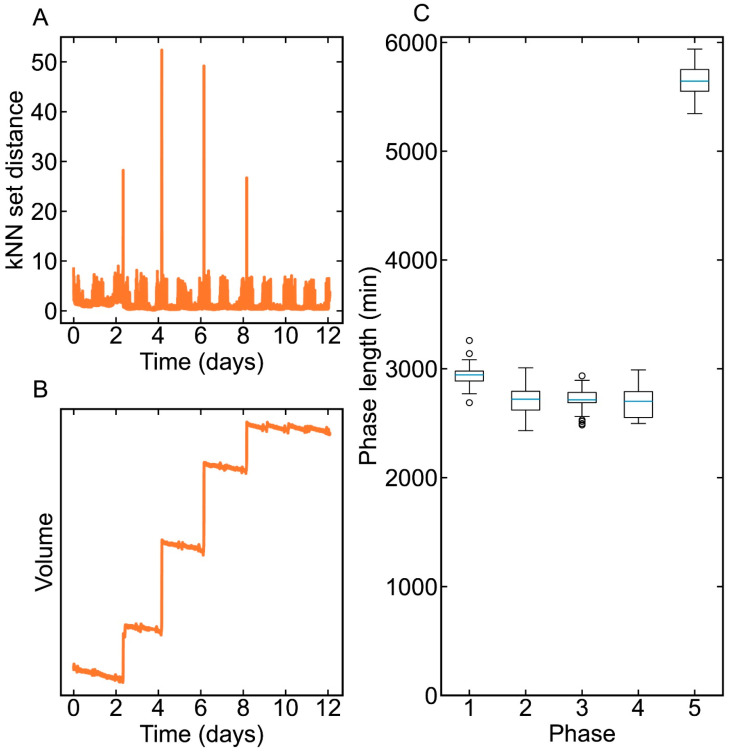
The *k*NN set distance (**A**) and the volume (**B**) of a typical batch, and the phase lengths of each batch in all batches (**C**).

The $d(x_{t})$ of a typical batch is shown in Figure 6A. It can be easily observed that four spikes appeared on Day 2, 4, 6, and 8, respectively. A high $d(x_{t})$ means that the samples at the corresponding moving window had high varieties. Therefore, the samples corresponding to the spikes were regarded as transient-phase samples and each batch could be divided into five phases by the *k*NN set rule. It can be also found that the spikes correspond to the volume changes, which were caused by the feeding of nutrients into the cell culture process. This shows that, in this case study, the changes in process characteristics were mainly caused by the feeding operations, instead of the temperature shift or other effects. In Figure 6C, the lengths of each phase in all batches are shown using a boxplot. The lengths of the first four phases were about 2800 min, which was nearly 2 days, while the length of the fifth phase was about 5600 min, i.e., nearly 4 days.

### 3.4. Feature Extraction and Fusion Result

#### 3.4.1. Reference Phase Selection

**Figure 7:**
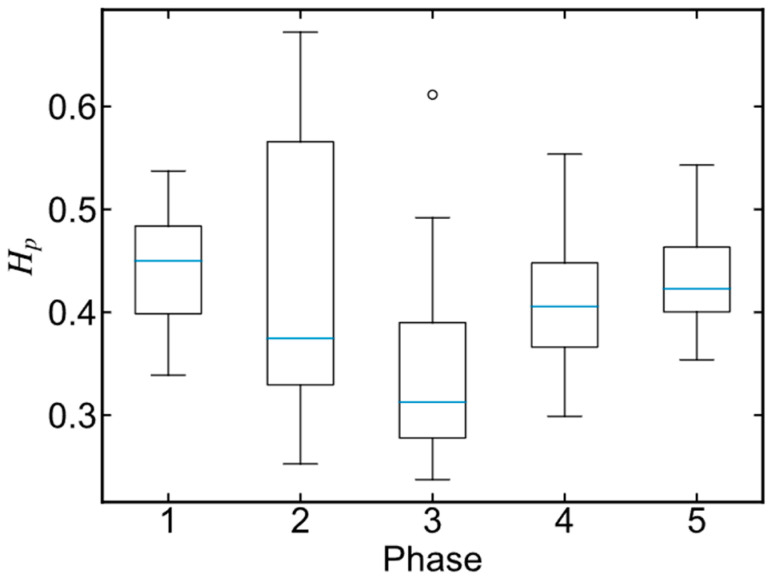
The distributions of the reference batch indicators, $H_{p}$, for all phases.

After the phases were divided, the features of each batch were extracted using the FDA method. The core of FDA fitting is to choose the same types and number of basis functions for each variable in each phase, and then the original process data are represented by a series of basis functions, which solves the uneven batch problem. To determine the number and type, the reference phases must be found as templates in advance. The indicator $H_{p}$ shown in Equation (8) combines the phase length and the trajectory smoothness as a measurement of the complexity of each phase. As described in Section 3.3, each batch was divided into five phases, so there were five reference phases. The $H_{p}$ indicator of each phase depicted in Figure 7 was in a wide range, meaning that different batches had high varieties in both lengths and smoothness. The phase with the highest $H_{p}$ was chosen as the reference phase.

#### 3.4.2. FDA Fitting Result

**Figure 8:**
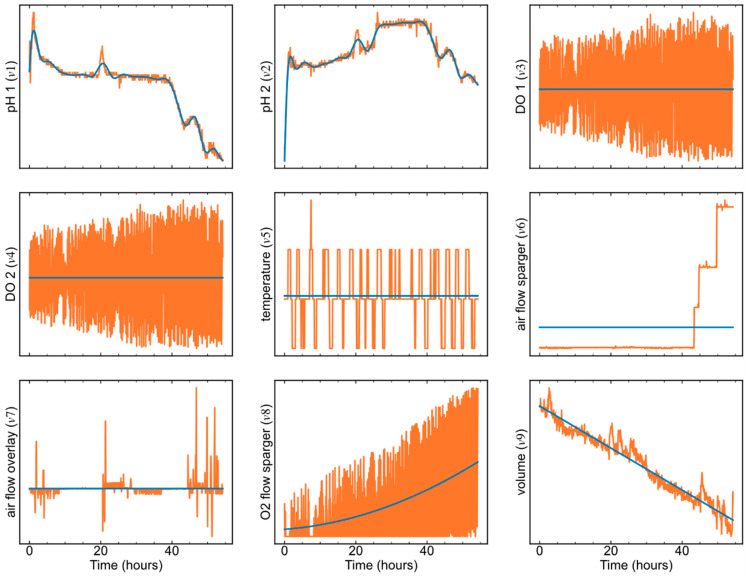
The inline process variable trajectories (in orange) and the corresponding fitted data (in blue) in the first phase.

In this step, the B-spline and polynomial basis functions were used to fit the process variables, as in Equation (10):

(10) $$x=\sum c_{i}B_{i,3}(t)\;x=\sum c_{i}x^{i-1}$$

where $B_{i,3}(t)$ is the *i*th degree-3 B-spline basis function, and $x^{i-1}$ is the *i*th polynomial basis function. It should be noted that if *n* polynomial basis functions are used, the highest degree of the functions is *n* − 1. Whether to use the B-spline or the polynomial basis function depends on the variable characteristics. To more clearly explain this, the variable trajectories and the fitted data in the first phase are shown in Figure 8. For *v*1 (pH 1) and *v*2 (pH 2), the adopted basis function was the B-spline function, because these two variables were relatively smooth and curved, having an obvious “functional characteristic”. For *v*3 (DO 1), *v*4 (DO 2), *v*5 (temperature), *v*6 (air flow sparger), and *v*7 (air flow overlay), these five variables did not have functional characteristics but were like step functions. Therefore, the basis function type for them was the degree-zero polynomial function, i.e., a horizontal line of the mean values, and the basis function number was 1. For *v*8 (O_2_ flow sparger), the trajectory was fitted using three polynomial basis functions, i.e., the quadratic polynomial. For *v*9 (volume), the variable trajectory was like a straight line; thus, it was appropriate to use the degree-1 polynomial basis function (the basis function number is 2) to fit *v*9.

**Figure 9:**
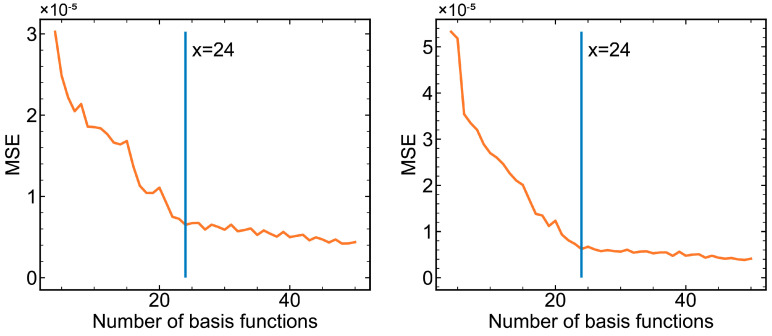
The relationship between MSE values and the number of B-spline basis functions. **Left**: *v*1; **right**: *v*2.

Once the basis function type is determined, the number of the B-spline basis functions needs to be optimized via the MSE of the original data and the fitted data. Again, taking *v*1 and *v*2 in the first phase as examples, the relationship between MSE values and the number of basis functions is shown in Figure 9. As more functions were employed to fit the original data, the MSE decreased sharply at first, then dropped slowly when the number of basis functions exceeded 24. Therefore, for *v*1 and *v*2 in the first phase, the B-spline function numbers needed to be set to 24 for each variable. Table 2 lists the basis function types and numbers for all variables in all phases.

After all inline variables were fitted via basis functions, the coefficients were extracted as the features of all batches. In this case, there were 368 basis functions used to fit all the inline variables in all phases, and consequently, the size of the inline variable feature matrix was 32 × 368. Then, the offline data were unfolded batch-wisely, and the size of the offline dataset was 32 × 84. Finally, the inline data feature matrix and the offline data were combined to form an extended feature matrix, the size of which was 32 × 452. Consequently, the original data containing about 7,361,280 sampling points and 84 offline data for each batch were replaced by the extended feature matrix. The feature extraction step resulted in a drastic reduction in data volume, simultaneously solving the uneven batching problem.

### 3.5. Cluster Comparing Result

Among the 32 batches, the data of the first 26 batches were initially collected and analyzed to find out the causes of productivity reduction via ccPCA, and then some decisions were made to improve productivity. The last six batches were validation batches, which were used to validate the data analysis results.

**Figure 10:**
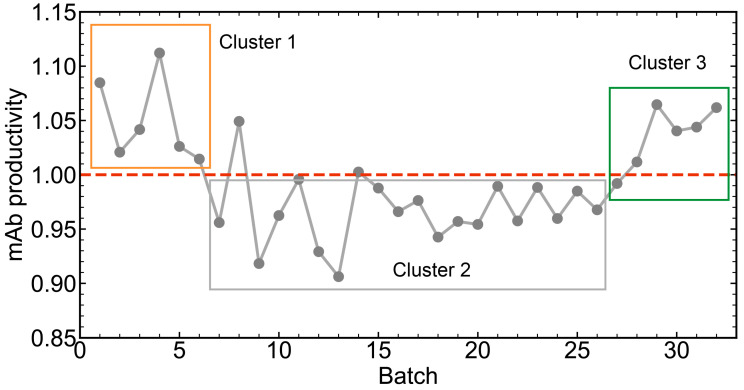
The mAb productivity of all batches. The batches with productivity below 1.0 were low-productivity batches, and the batches with productivity above 1.0 were high-productivity batches.

The mAb productivity of all batches is shown in Figure 10, and the real productivity values were divided by a certain number for data confidentiality. The batches with productivity higher than 1.0 were considered high-productivity batches and the batches with productivity lower than 1.0 were classified as low-productivity batches. As can be seen, the first six batches had high productivity and were labeled as Cluster 1. Then, the productivity decreased to below 1.00, and these batches were labeled as Cluster 2. The validation batches were labeled as Cluster 3. The average productivity of the batches in Cluster 1 was 1.050, and the average productivity of the batches in Cluster 2 was 0.961. The difference in mAb productivity between Cluster 1 and Cluster 2 was 0.089, which represented a loss of 8.48%. A one-tailed Student *t*-test was performed to compare the productivities of Cluster 1 and Cluster 2, and the *p*-value was less than 0.05. The results showed that the mAb productivity of Cluster 1 was significantly higher than that of Cluster 2.

**Figure 11:**
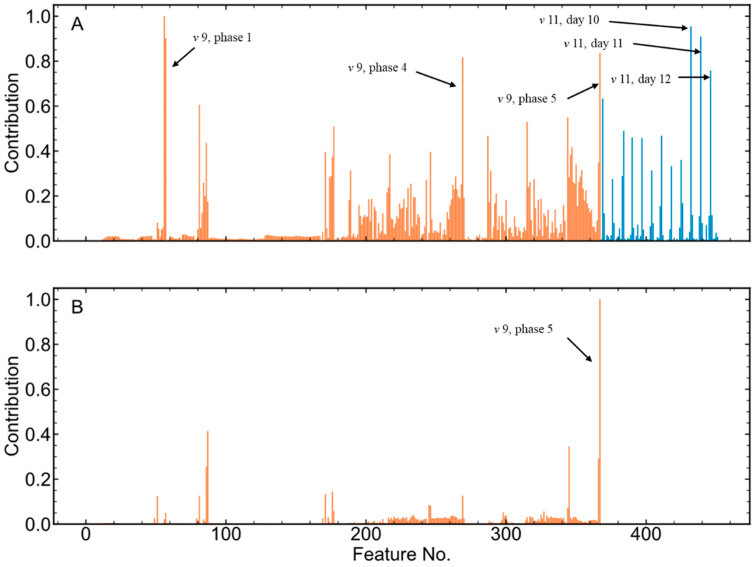
The contribution of each feature to the differences between Cluster 1 and Cluster 2 (**A**) and the contribution of each feature to the differences between Cluster 3 and Cluster 2 (**B**). The bars in orange represent the inline data features and the bars in blue represent the offline data features.

The trends of inline and offline variables are shown in Figures S1 and S2. To identify the causes of such a productivity reduction, ccPCA was applied to the extended feature matrix to compare Cluster 1 with Cluster 2. The union set of these two clusters was treated as the entire dataset, ***E***, and Cluster 2 was set as the background dataset, ***R***, in Equation (6). A ccPCA model was then built to discover the differences between the two clusters. As shown in Figure 11A, the contributions of all the features to the difference between Cluster 1 and Cluster 2 were calculated using the loadings of the first cPC. The features of two variables were found to have high contributions: *v*9 (volume) and *v*11 (cell viability). More specifically, the features of *v*9 in Phase 1, 4, and 5 and *v*11 on Day 10, 11, 12 contributed to the differences the most.

**Figure 12:**
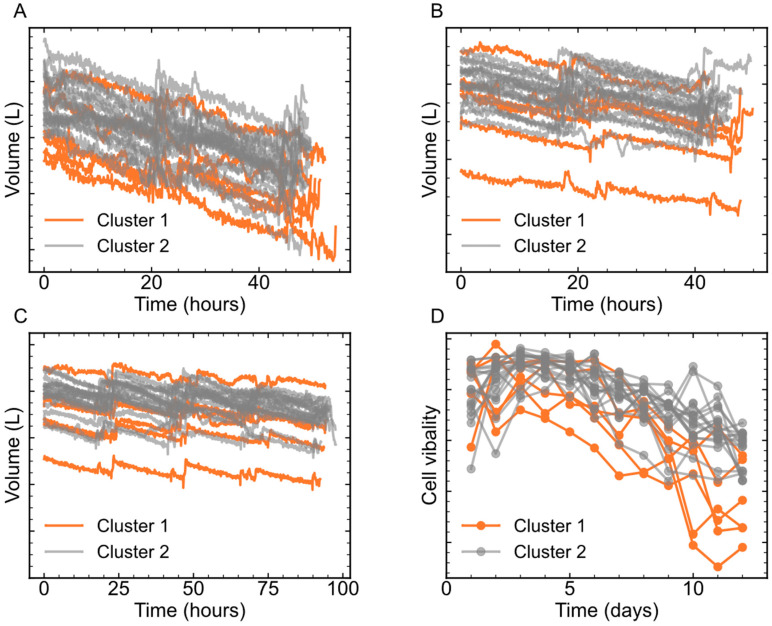
The trajectories of *v*9 and *v*11 in Cluster 1 and Cluster 2. (**A**) *v*9 in Phase 1; (**B**) v9 in Phase 4; (**C**) *v*9 in Phase 5; (**D**) *v*11 on different days.

To further identify the causes of the productivity difference between Cluster 1 and Cluster 2, the trajectories of *v*9 and *v*11 of Cluster 1 and Cluster 2 were carefully checked, as shown in Figure 12. The *v*9 in Cluster 1 was slightly lower than that in Cluster 2, but most trajectories overlapped, while *v*11 on the last 3 days in Cluster 1 was much lower. The results meant that lower cell viability might be beneficial for mAb productivity in this case, which went against expectations. In many cases, lower cell viability would lead to fewer cells producing mAbs, and thus the productivity would indeed decrease. However, the contradictory result in this case indicated that fewer cells were able to produce more mAbs. The reason for this might be that on the last several days of the cell culture process, the nutrients were not sufficient to support cells to express adequate proteins. If the cell viability was high, more nutrients would be consumed for cell growth instead of protein expression. Therefore, a reasonable decision would be to feed more cell culture medium into the cell culture process.

**Figure 13:**
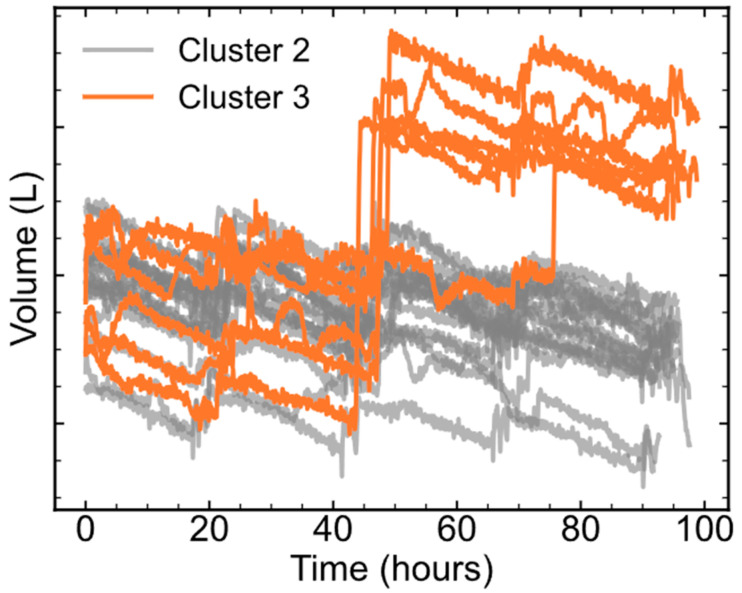
The trajectories of *v*9 in Phase 5 in Cluster 2 and Cluster 3.

According to the comparison between Cluster 1 and Cluster 2, additional feeding of cell culture medium in the last stage of the cell culture process was carried out in the batches of Cluster 3. Consequently, the productivity of Cluster 3 increased to about 1.05, as shown in Figure 10. Again, a one-tailed Student *t*-test was also performed to compare the productivities of Cluster 2 and Cluster 3, and the *p*-value was less than 0.05. The results showed that the mAb productivity of Cluster 3 was significantly higher than that of Cluster 2. To investigate the differences between Cluster 2 and Cluster 3, ccPCA was also applied. Similarly, the contribution of each feature to the differences between Cluster 3 and Cluster 2 is shown in Figure 11B. The features of *v*9 in Phase 5 contributed the most, while other features’ contributions were much lower. Thus, the biggest difference between Cluster 3 and Cluster 2 was in *v*9 in Phase 5. The trajectories of *v*9 in Phase 5 shown in Figure 13 indicated that in Cluster 3, there was a sudden rise at the 40th hour (on Day 10), and this corresponded to additional cell culture medium being fed into the process. In other words, the additional feeding of cell culture medium into the process on Day 10 did improve mAb productivity, which proved the results obtained previously.

Combining the above results, the cause leading to the mAb productivity reduction was the inadequate amount of nutrients to support protein expression. The high cell viability in Cluster 2 meant more nutrients were used for cell growth instead of protein expression, and thus, the additional feeding in Cluster 3 provided adequate nutrients for cells to produce more mAbs. Therefore, it is suggested that researchers feed additional nutrients into the process on Day 10 for higher mAb productivity in the future. The reason for the inadequate amount of nutrients may have been that the amount of nutrients was optimized at small scales (laboratory scales and pilot scales) but was not optimal for the commercial-scale cell culture process.

## 4. Conclusions

In this study, a data-driven approach was proposed to exploit inline and offline data from a commercial-scale cell culture process to discover the causes of mAb productivity reduction. The approach consisted of four steps, namely data preprocessing, phase division, feature extraction and fusion, and cluster comparison. In the data preprocessing step, the data quality issues were solved for further analysis. In the phase division step, the moving window *k*NN rule was applied to detect the transient phases and segment the whole batch into several stable phases. In the feature extraction step, each inline variable was fitted using the FDA method, and the basis function coefficients were extracted to form the feature matrix, which was combined with the offline data. In the cluster comparing step, the contributions of all features to the difference between high-productivity batches and low-productivity batches were calculated to identify the causes of the productivity reduction. With this approach, the inline and offline data were fully analyzed to discover the causes of the mAb productivity reduction.

A case study of the commercial-scale cell culture process was provided to verify the effectiveness of the approach. Data from 35 batches were collected, and each batch contained 71 inline variables and 7 offline variables. After the data preprocessing step, three batches and 62 inline variables were discarded. The features of the remaining data were extracted via the FDA method, and the size of the extended feature matrix was 32 × 452. Finally, the differences between high-productivity batches and low-productivity batches were compared, and the result was proven via validation batches. The results showed that the cause of the productivity reduction was the high cell viability, which led to an inadequate amount of nutrients for protein expression. This data-driven approach can help biopharmaceutical companies make timely and accurate decisions, which in turn can improve profits and cut costs.

This approach has the potential to be applied to other pharmaceutical manufacturing processes, which also have multistage and uneven batch length problems. The limitations of the approach are that the standards for selecting the basis function number for FDA and for selecting the moving window *k*NN parameters are subjective. These issues will be discussed in future studies.

## Figures and Tables

**Table 1:** The process variables after data preprocessing.

No.	Inline Process Variables	Unit	No.	Offline Process Variables	Unit
1	pH 1	―	10	VCD	10^6^·mL^−1^
2	pH 2	―	11	cell viability	%
3	DO 1	%	12	average diameter of cells	μm
4	DO 2	%	13	cell agglomeration ratio	%
5	temperature	°C	14	pH	―
6	air flow sparger	L·min^−1^	15	glucose concentration	g·L^−1^
7	air flow overlay	L·min^−1^	16	lactate concentration	g·L^−1^
8	O_2_ flow sparger	L·min^−1^			
9	volume	L			

**Table 2:** The basis function types and numbers for all variables in all phases.

Variable No.	Phase 1		Phase 2		Phase 3		Phase 4		Phase 5	
	Type *	Number	Type	Number	Type	Number	Type	Number	Type	Number
1	B	24	B	9	B	40	B	5	B	8
2	B	24	B	11	B	40	B	5	B	8
3	P	1	P	1	P	1	B	28	B	28
4	P	1	P	1	P	1	B	28	B	28
5	P	1	P	1	P	1	P	1	P	1
6	P	1	P	1	P	1	P	1	P	1
7	P	1	P	1	P	1	P	1	P	1
8	P	3	P	3	P	3	B	22	B	20

* B: B-spline basis function; P: polynomial basis function.

## Data Availability

The datasets presented in this article are not readily available because the datasets involve trade secrets and are not allowed to be shared publicly.

## Supplementary Materials

The following supporting information can be downloaded at https://www.mdpi.com/article/10.3390/pharmaceutics16081082/s1, Figure S1: The trajectories of inline variables in Cluster 1 and Cluster 2; Figure S2: The trajectories of offline variables in Cluster 1 and Cluster 2.
