# Supplementary material for: A Data-Driven Approach for Leveraging Inline and Offline Data to Determine the Causes of Monoclonal Antibody Productivity Reduction in the Commercial-Scale Cell Culture Process
Source: Pharmaceutics. 2024 Aug 17;16(8):1082. doi: 10.3390/pharmaceutics16081082 (PMC11359819; doi:10.3390/pharmaceutics16081082)
Supplement: Supplementary file 1 [file pharmaceutics-16-01082-s001.zip › pharmaceutics-3081484-supplementary.pdf]

A Data-Driven Approach for Leveraging Inline and Offline Data to Determine the Causes of Monoclonal Antibody Productivity Reduction in the Commercial-Scale Cell Culture Process

Sheng Zhang, Hang Chen, Yuxiang Wan, Haibin Wang and Haibin Qu

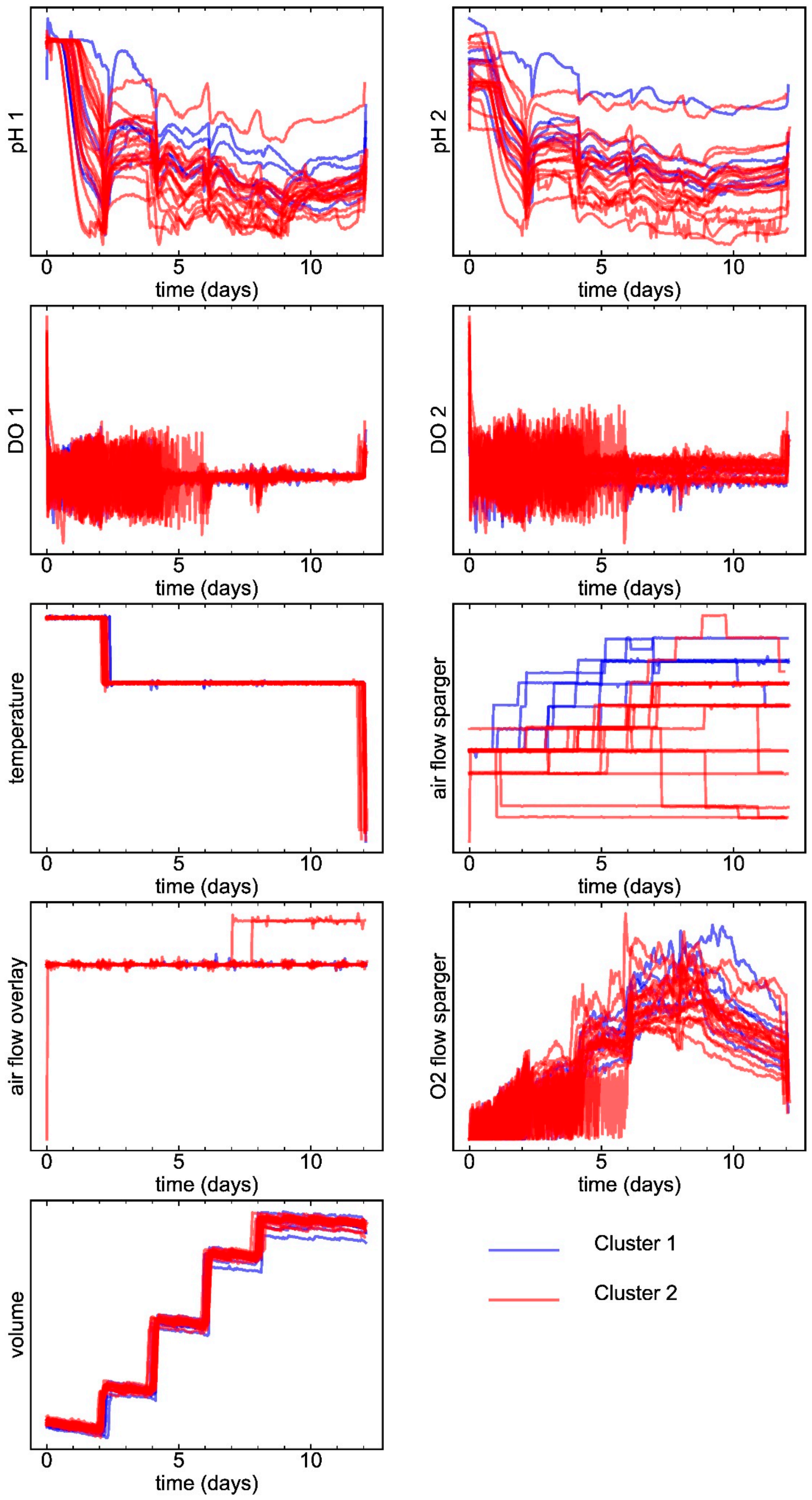

Figure S1. The trajectories of inline variables in Cluster 1 and Cluster 2.

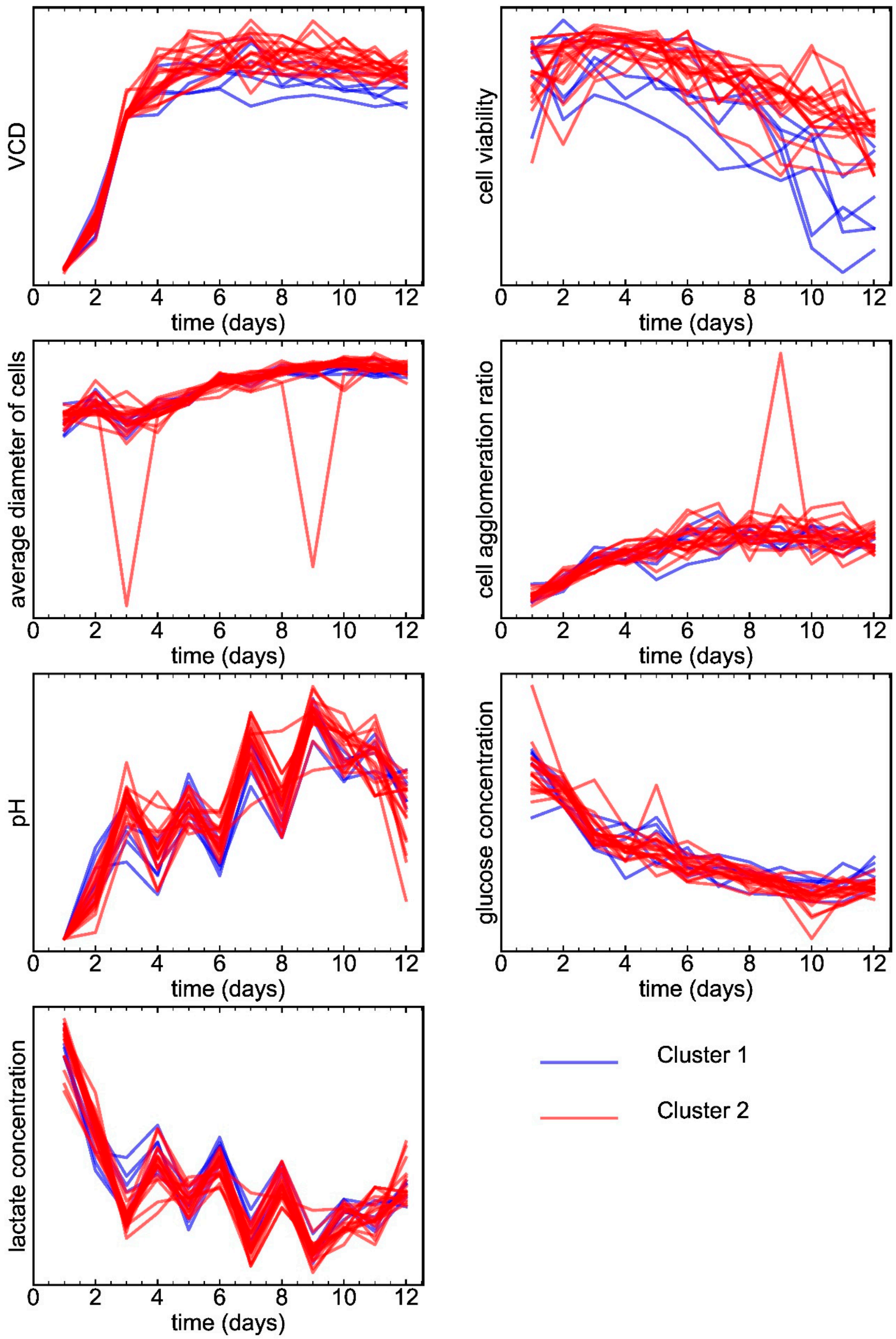

Figure S2. The trajectories of offline variables in Cluster 1 and Cluster 2.
